# Sigmoid volvulus and diabetes mellitus

**DOI:** 10.12669/pjms.39.3.7309

**Published:** 2023

**Authors:** Esra Disci, Rifat Peksoz, Sabri Selcuk Atamanalp

**Affiliations:** 1 Esra Disci, MD, Associate Professor Department of General Surgery, Faculty of Medicine, Ataturk University, Erzurum, Turkey; 2 Rifat Peksoz, MD, Assistant Professor Department of General Surgery, Faculty of Medicine, Ataturk University, Erzurum, Turkey; 3 Prof. Sabri Selcuk Atamanalp, MD, Department of General Surgery, Faculty of Medicine, Ataturk University, Erzurum, Turkey

**Keywords:** Sigmoid volvulus, Diabetes mellitus, Comorbidity

## Abstract

**Objectives:**

Diabetes mellitus (DM) complicates about 15.7% of sigmoid volvulus (SV) cases. However, the pathophysiology of this relation is still unclear. Our objective was to evaluate the association of DM and SV.

**Methods:**

The clinical data of 1,051 patients treated in Ataturk University Faculty of Medicine during 56 years between June 1966 and July 2022 were considered. The records of 612 cases (58.2%) were evaluated retrospectively till June 1986, while 439 (41.8%) were investigated prospectively thereafter. To obtain the worldwide data, an electronic search of the last 56-years’ literature (from 1967 to date) was performed in Web of Science and PubMed databases.

**Results:**

DM was statistically higher in SV patients than of general population (15.7% vs. 8.3%, p<0.001). Conversely, SV and DM co-occurrence was statistically lower in our series than of world-wide data (2.9% vs. 15.7%, p<0.001). In our series, SV and DM comorbidity was statistically higher in elders that that of children (3.9% vs. 0.0%, p<0.05). Although sigmoid gangrene was more common in DM patients when compared with that of total, the difference was not statistically significant (42.9% vs. 27.4%, p>0.05). Conversely, the mortality rate was statistically higher in DM cases than that of no diabetics in SV (28.6% vs. 7.8%, p<0.001).

**Conclusion:**

Although the pathophysiology of SV and DM comorbidity is still relatively unexplainable, our study suggests that DM worsens the prognosis of SV. For this reason, early diagnosis and proper treatment have great importance in such patients.

## INTRODUCTION

The relationship between sigmoid volvulus (SV) and diabetes mellitus (DM) is a relatively complex as well as an uninvestigated subject in literature.^1^,^2^ Although some authors mentioned about the co-occurrence of SV and DM as a theoretical information in their articles and some others wrote about DM briefly when they declared the morbidities in their SV series in past, an electronic data search of the last 56-years’ literature (from 1967 to date) in Web of Science^1^ and PubMed^2^ databases revealed only two searches on the pathophysiology of this relation.^3^,^4^ For this reason, we want to discuss the association of SV and DM together with our 1,051-case SV experience obtained in 56 years duration (from June 1966 to July 2022), which is the most comprehensive monocenter SV series.^5^

## METHODS

In Ataturk University Faculty of Medicine, total 1,051 SV patients were treated during 56 years between June 1966 and July 2022. The records of 612 cases (58.2%) were evaluated retrospectively till June 1986, while 439 (41.8%) were investigated prospectively thereafter. For each case, age, gender, comorbidity including DM, sigmoid viability, and mortality were noted. In the clinical practice, following diagnosis and resuscitation, endoscopic decompression was applied in uncomplicated cases (without peritoneal irritation or bowel gangrene findings), whereas complicated cases (with above mentioned adverse conditions or unsuccessful endoscopic decompression) treated with emergency surgery. On the other hand, DM and SV comorbidity was investigated in Web of Science^1^ and PubMed^2^ databases under the heads of ‘sigmoid volvulus’ and ‘sigmoid volvulus and diabetes mellitus’. The obtained data were evaluated together with the results of our series.

Statistical analyses were performed by using SPSS v22.0 package (IBM Corporation, Armonk, New York, United States). Data were expressed as numerical variables or percentages for categories. Categorical variables were compared by using Chi-Square, Linear-by-Linear association, and Fisher exact tests. Significance level was set up p<0.05. Ethical approval was obtained from the institutional review board, Ethical Committee of Ataturk University Faculty of Medicine (22/88). Written informed consent for scientific research was obtained from all participants.

## RESULTS

Our results on SV and DM relation are shown in Table-I while Table-II presents world-wide DM prevalence in SV patients reported in certain publications. DM was statistically more common in SV patients when compared with that of DM prevalence in regional population (15.7% vs. 8.3%,^6^ Chi-square test, p<0.001). In our series, 28 (2.9%) of 950 patients, in which the records were obtained, were diabetic. When compared with that of DM prevalence in world-wide SV cases, ours was relatively low (2.9% vs. 15.7%, Chi-square test, p<0.001). Among 950 patients, SV was seen in 12 (1.3%) children, 430 (45.3%) adults, and 508 (53.5%) elders (over the age of 60) with a mean age of 59.5 years. In SV group, DM wasn’t present in childhood (0.0%), while it was determined in a rate of 1.9% (eight patients) in adults and 3.9% (20 patients) in cases over 60 years of age. In statistical analysis, DM complicating SV was found higher in elders than that of children (Linear-by-Linear association test, p<0.05), while other comparisons were statistically similar (Linear-by-Linear association test, p>0.05). Although sigmoid gangrene was more common in DM group (12/18, 42.9%) when compared with that of total (288/1,051, 27.4%), the difference was not statistically significant (Chi-square test, p>0.05). Conversely, the mortality rate was statistically higher (Ficher exact test, p<0.001) in DM patients (8/28, 28.6%) than that of nondiabetic SV cases (80/1,023, 7.8%).

**Table-I:** The results of the evaluated criteria and the statistical analyses.

Criterion	Data				Statistical analyses
Diabetes mellitus in sigmoid volvulus	*Children*	*Adults*		*Elders*	Linear-by-Linear association p:0.049 children-elders p>0.05 others
	0/12 (0.0%)	8/430 (1.9%)		20/508 (3.9%)
Sigmoid gangrene	*Diabetes mellitus*		*Total*		Chi-Square p:0.072
	12/28 (42.9%)		288/1,051 (27.4%)	
Mortality	*Diabetes mellitus*		*Nondiabetic*		Ficher exact p<0.001
	8/28 (28.6%)		80/1,023 (7.8%)	

**Table-II:** Diabetes mellitus complicating sigmoid volvulus in the literature.

Author	Year	Sigmoid volvulus	Diabetes mellitus	%
Berenyi and Schwarz^3^	1967	13	5	38.5
Raveenthiran^4^	2003	86	10	11.6
Bhatnagar et al.^7^	2004	76	2	2.6
Tan et al.^8^	2010	71	13	18.3
Gupta et al.^9^	2011	72	11	15.3
Atamanalp^10^	2013	938	26	2.8
Halabi et al.^11^	2014	19,220	3,306	17.2
Dolejs et al.^12^	2018	2,538	307	12.1
Althans et al.^13^	2019	1,514	160	10.6
Uylas and Kayaalp^14^	2020	134	5	3.7
Tankel et al.^15^	2021	58	9	15.5
Surek et al.^16^	2021	73	30	41.1
Emna et al.^17^	2022	67	12	17.9
Moro-Valdezate et al.^18^	2022	92	22	23.9

Total		24,952	3,918	15.7

## DISCUSSION

Although DM prevalence in regional population is stated as 8.3%,^6^ when case reports and small case series are excluded, the rate of the co-occurrence of SV and DM is reported to be between 2.6% and 41.1% (mean 15.7%) in the literature (Table-II).^3^,^4^,^7^-^18^ Halabi et al.^11^ reported 17.2% of DM rate in the largest multicenter study including 19,220 SV cases. Similarly, Dolejs et al.^12^ and Althans et al.^13^ demonstrated 12.1% and 10.6% of DM incidences in relatively large multicenter SV series including 2,538 and 1,514 cases, respectively.

The relationships between SV and some clinical entities including chronic constipation, mental disability, Alzheimer’s disease, Parkinson’s disease, and Hirschsprung’s disease are defined well enough.^19^-^22^ However, SV and DM relation, particularly its pathophysiology is not clear enough.^1^,^2^ The first report on DM complicating SV was published in 1967 by Berenyi and Schwarz.^3^ In this report, the authors presented five DM cases (38.5%) among 13 SV patients and they hypothesized that diabetic mesenteric visceral neuropathy leads up to sigmoid megacolon, which is an anatomical predisposing factor in the development of SV.

In 2003, Raveenthiran^4^ wrote a preliminary report including 10 cases (11.6%) with DM among total 86 SV patients. In this report, although the author didn’t give a detailed information about the pathophysiology, he believed that the co-occurrence of SV and DM is not mere coincidence, and these diseases may have similar etiological factors. Since that time, although some authors gave some information about the incidence of DM in SV,^7^-^18^ unfortunately, no other paper was published on the pathophysiology of this relation.^1^,^2^

Although DM prevalence in SV patients is relatively higher than that of DM prevalence in general population both on average and in large SV series, it is still not clear that whether there is a correlation between SV and DM, and if any, it is a cause and effect relation or a simple coincidence. On this topic, the diabetic vasculapathy hypothesis of Berenyi and Schwarz^3^ seems as reasonable. However, in our opinion, if and only new studies including comparative histopathological examination of the resected sigmoid colon as well as sigmoid mesentery materials in surgically treated SV patients with or without DM may clear up this subject. Although DM is not directly correlated with dolichosigmoid at least for now,^19^ if this theory is reliable, the relatively low DM rate in our SV series (2.9%) when compared with that of the literature (mean 15.7%) may be explained by the dominant effects of high-fiber diet habit and live at high altitude on the development of sigmoid megacolon.

Another important factor affecting this relatively low rate may be partial retrospective nature of our study. However, when it comes to DM and SV relation, the similar etiological factors assessment of Raveenthiran^4^ may also not be considered. In our series, both increased SV and DM rates at later ages may support this idea. In the end, no matter which theory is correct, our series demonstrates that DM increases mortality risks in SV patients.

**Fig.1:**
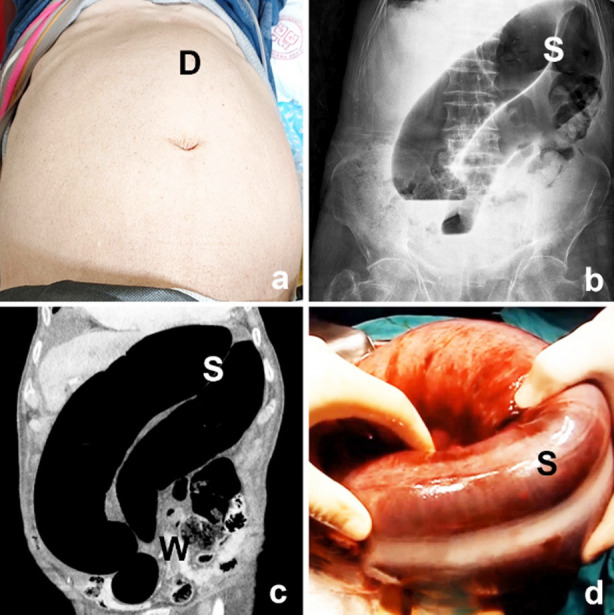
Clinical, radiological, and operative findings in diabetic patients with sigmoid volvulus. a. Clinical appearance (D: left-sided abdominal distention). b. Abdominal x-ray image (S: dolichocolic sigmoid colon). c. Abdominal coronal computed tomography image (S: dolichocolic sigmoid colon, W: whirled sigmoid mesentery). d. Operative appearance (S: dolichocolic sigmoid colon).

Irrespectively of the incidence of SV and DM association, the clinical presentation and diagnosis of SV in diabetic patients doesn’t differ than that of nondiabetics. Abdominal pain/tenderness, distention (Fig.1a), and obstipation/constipation are the main clinical features. Abdominal x-ray radiography demonstrates dolichocolic sigmoid colon (Fig.1b), while computed tomography is highly diagnostic with similar findings in addition to whirl in sigmoid mesentery (Fig.1c).^5^,^10^,^14^,^21^

Regarding the treatment strategy, the basic rules are similar, which consist of endoscopic decompression in uncomplicated patients and surgical treatment in complicated cases,^5^,^7^-^9^,^11^,^12^,^15^-^18^,^20^,^21^,^23^-^25^ in whom dolichocolic sigmoid colon is the mean laparotomy feature (Fig.1d). However, it mustn’t be forgotten that, DM increases mortality risks in SV patients, as was demonstrated in our series. For this reason, early detection and effective resuscitation followed by emergency endoscopic or surgical treatment is essential.

### Limitations:

Limited number of cases in some subgroups, partial retrospective evaluation, and long study period are probable limitations of this study. However, the limitation of SV incidence frequency obligates to use both retrospective evaluation and long-term period to obtain relatively sufficient material.

## CONCLUSIONS

DM complicates about 2.6% - 41.1% of SV cases. It is not clear that who and when will definitely explain the presence as well as the pathophysiology of this association, but this study suggests that DM increases mortality risk in SV. For this reason, practitioners must currently be careful to diagnose and treat such patients.

### Authors Contribution:

**ED, SSA:** Data collection, manuscript writing, revision of the final draft.

**RP:** Data collection, revision of the final draft.

**SSA:** is responsible for responsible and accountable for the accuracy or integrity of the work.
